# Supplementary material for: Glycogen Storage Disease Phenotypes Accompanying the Perturbation of the Methionine Cycle in NDRG3-Deficient Mouse Livers
Source: Cells. 2022 May 4;11(9):1536. doi: 10.3390/cells11091536 (PMC9103136; doi:10.3390/cells11091536)
Supplement: Supplementary file 1 [file cells-11-01536-s001.zip › cells-1641670-supplementary.pdf]

Figure S1

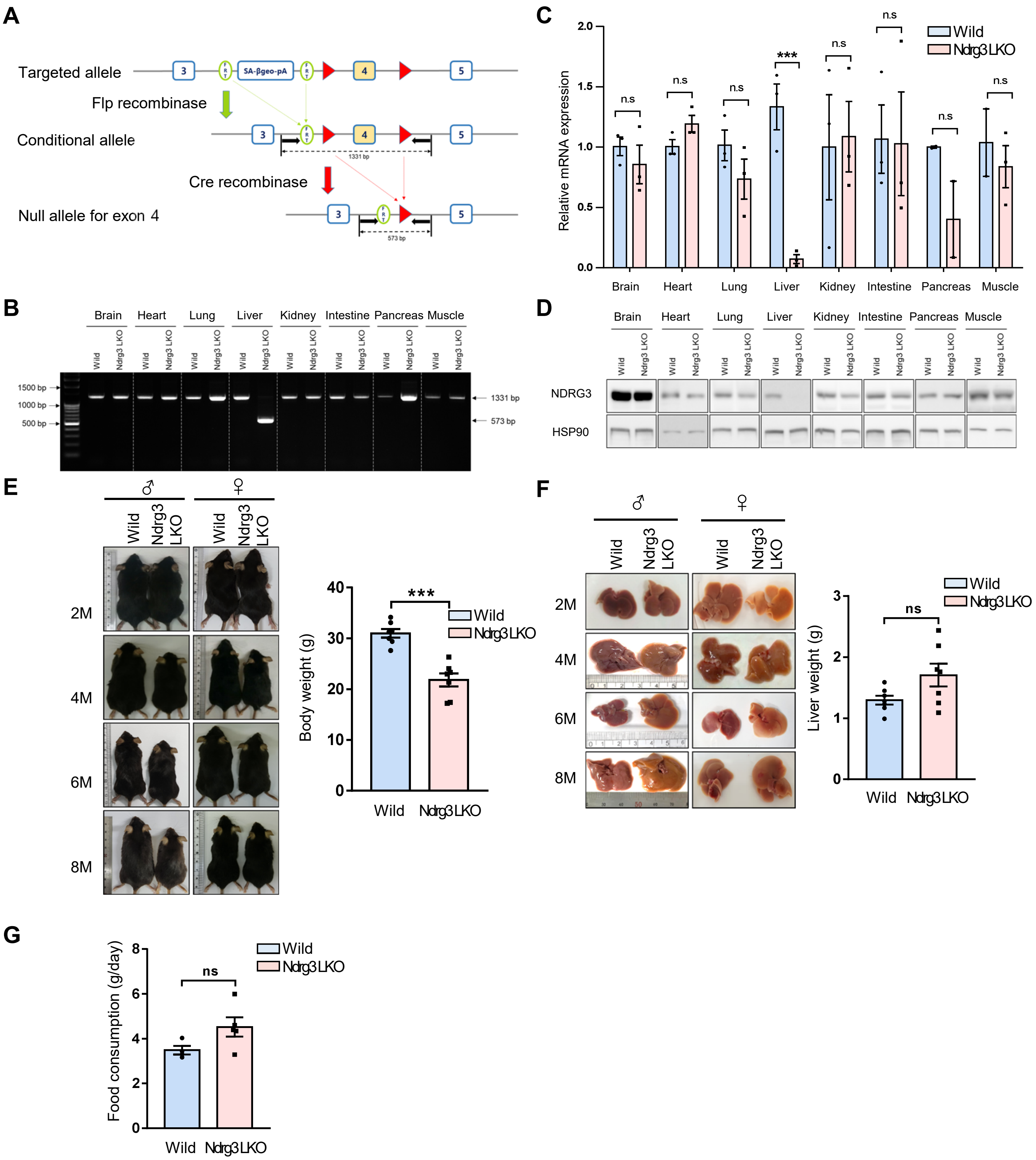

A
Targeted allele
Flp recombinase
Conditional allele
Cre recombinase
Null allele for exon 4
SA-βgeo-pA
1331 bp
573 bp
B
Brain Heart Lung Liver Kidney Intestine Pancreas Muscle
Wild
Ndrg3 LKO
1500 bp
1000 bp
500 bp
1331 bp
573 bp
C
Relative mRNA expression
n.s
***
Wild
Ndrg3 LKO
Brain Heart Lung Liver Kidney Intestine Pancreas Muscle
D
NDRG3
HSP90
E
♂ ♀
2M
4M
6M
8M
Body weight (g)
***
F
Liver weight (g)
ns
G
Food consumption (g/day)
ns

Figure S1. Generation of liver-specific KO mouse for *Ndrg3* gene and gross examination of pathologic phenotypes. (A) Scheme for the generation of mice harboring liver-specific deletion allele of *Ndrg3*. Exon 4 region of *Ndrg3* gene flanked by loxP sites (red arrowheads) was excised by albumin-Cre recombination system. Black arrows indicate the primer target sites for genomic DNA amplification to verify genetic deletion. (B) Liver-specific deletion of *Ndrg3* detected at the genomic DNA level via PCR analysis. Black arrows indicate the primer target size (wild length = 1331bp, *Ndrg3* knockout length = 573bp). (C) Quantitative real-time RT-PCR (qRT-PCR) analysis to verify liver-specific deletion of *Ndrg3* (n=2-3 per group). *Ndrg3* expression was normalized using *Gapdh* as the reference. (D) Verification of liver-specific NDRG3 deficiency at the protein level by a western blot analysis. (E) Representative images for the gross appearance of wild-type and NDRG3-deficient mice at increasing ages. Both male and female mice are shown. (F) Representative gross images for liver tissues from wild-type and NDRG3-deficient mice. (G) Daily food consumption by wild-type (n= 4) and Ndrg3 LKO (n= 5) mice. Males at 10-12 weeks of age were fasted overnight, and food weights (g) before and after refeeding were differentially calculated. Data are presented as mean ± SEM. *p*-values were calculated using two-tailed Student's *t*-test (C,E,F,G). ***$p < 0.001$ *vs* wild mice.

Figure S2

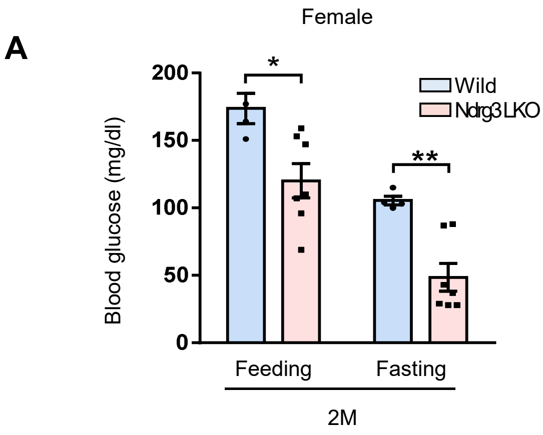

Figure S2. Hypoglycemia in female mice with liver-specific NDRG3 abrogation. (A) Blood glucose levels of 2-3-month-old, wild-type (n=4) and Ndrg3 LKO (n=7) female mice determined *ad libitum* (Feeding) or after overnight fasting (Fasting). *p < 0.05, **p < 0.01.

Figure S3

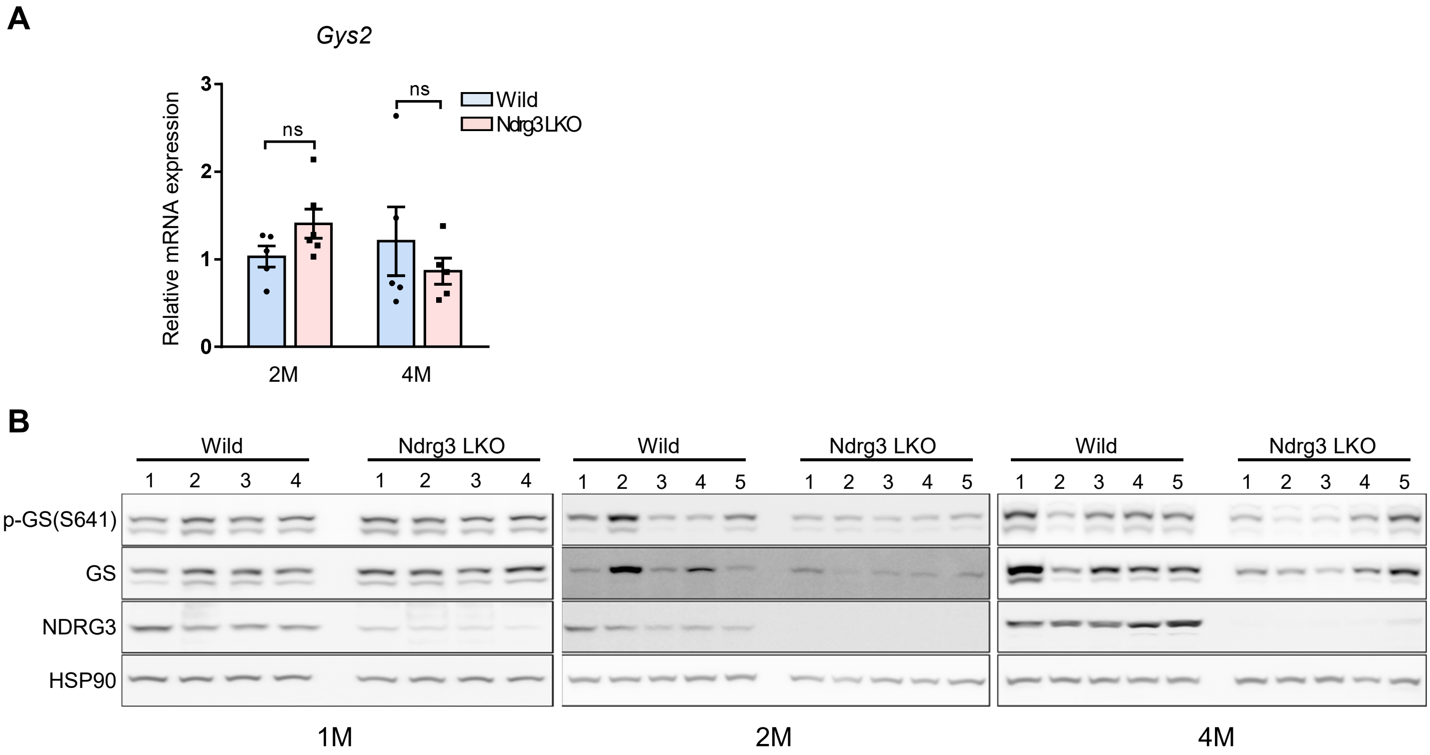

Figure S3. Intact glycogen biosynthesis capacity in Ndrg3 LKO mice. (A) qRT-PCR analysis of *Gys2* gene expression in wild-type and Ndrg3 LKO mouse livers at 2 or 4 months of age (n=5 per group). Mice were fasted overnight and then refed for 6 h before being subject to the gene expression analysis. Gene expression was normalized using *Gapdh* as the reference. (B) Western blot analysis for GS in wild-type and Ndrg3 LKO mouse livers at increasing ages (n=4-5 per group).

Figure S4

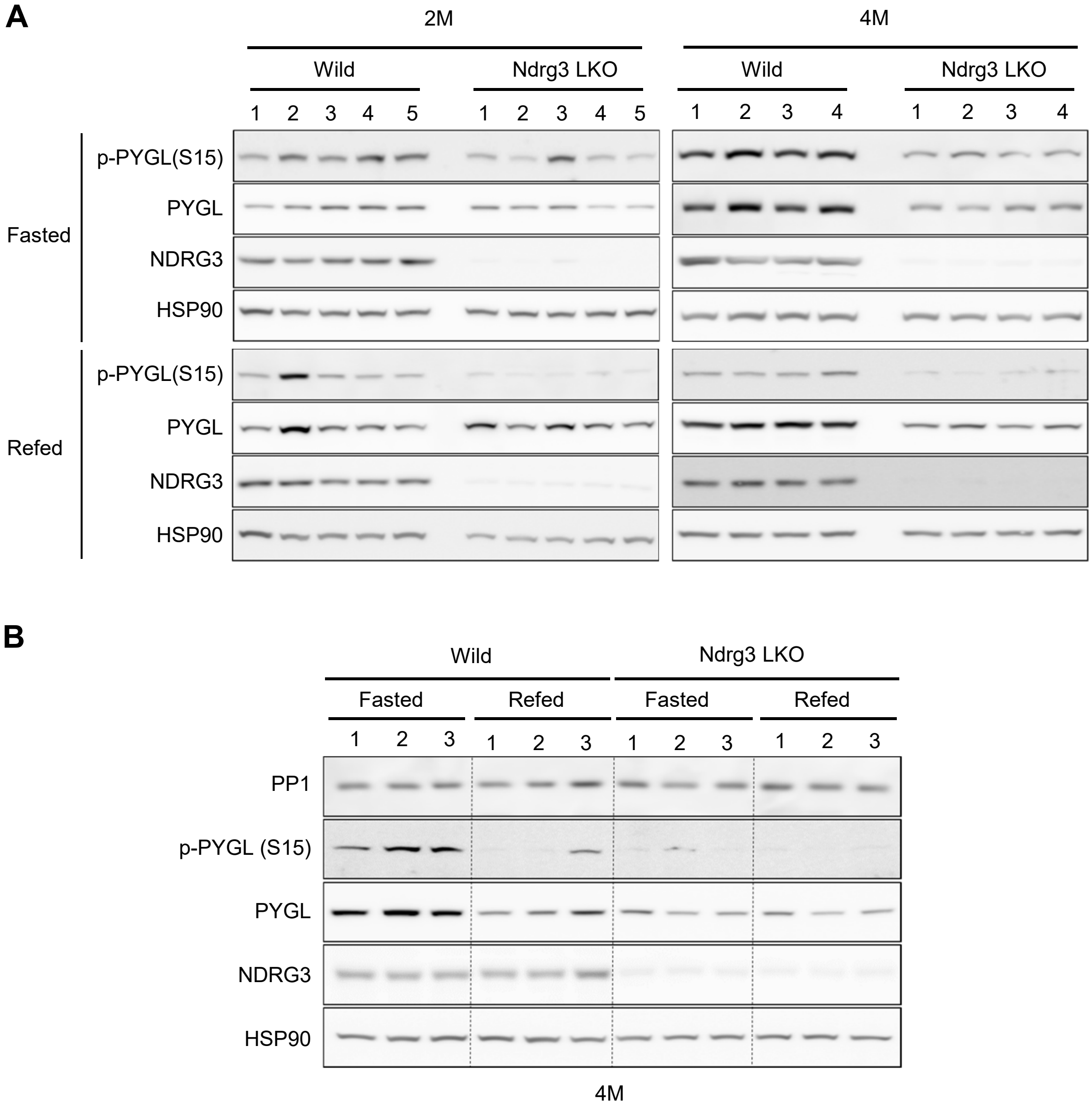

Figure S4. Dysregulation of PYGL expression and activity in Ndrg3 LKO livers. (A) Western blot analysis of PYGL and p-PYGL(S15) expression in wild-type and Ndrg3 LKO mouse livers at the age of 2 months (n=5 per group) or 4 months (n=4 per group). Mice were fasted for 15h or refed for additional 6h before being subject to the western blot analysis. (B) Western blot analysis of protein phosphatase 1 (PP1) expression in 4-month-old wild-type and Ndrg3 LKO mice (n=3 per group). Expression of PYGL and p-PYGL(S15) is also shown as the control for possible PP1-mediated dephosphorylation activity.

Figure S5

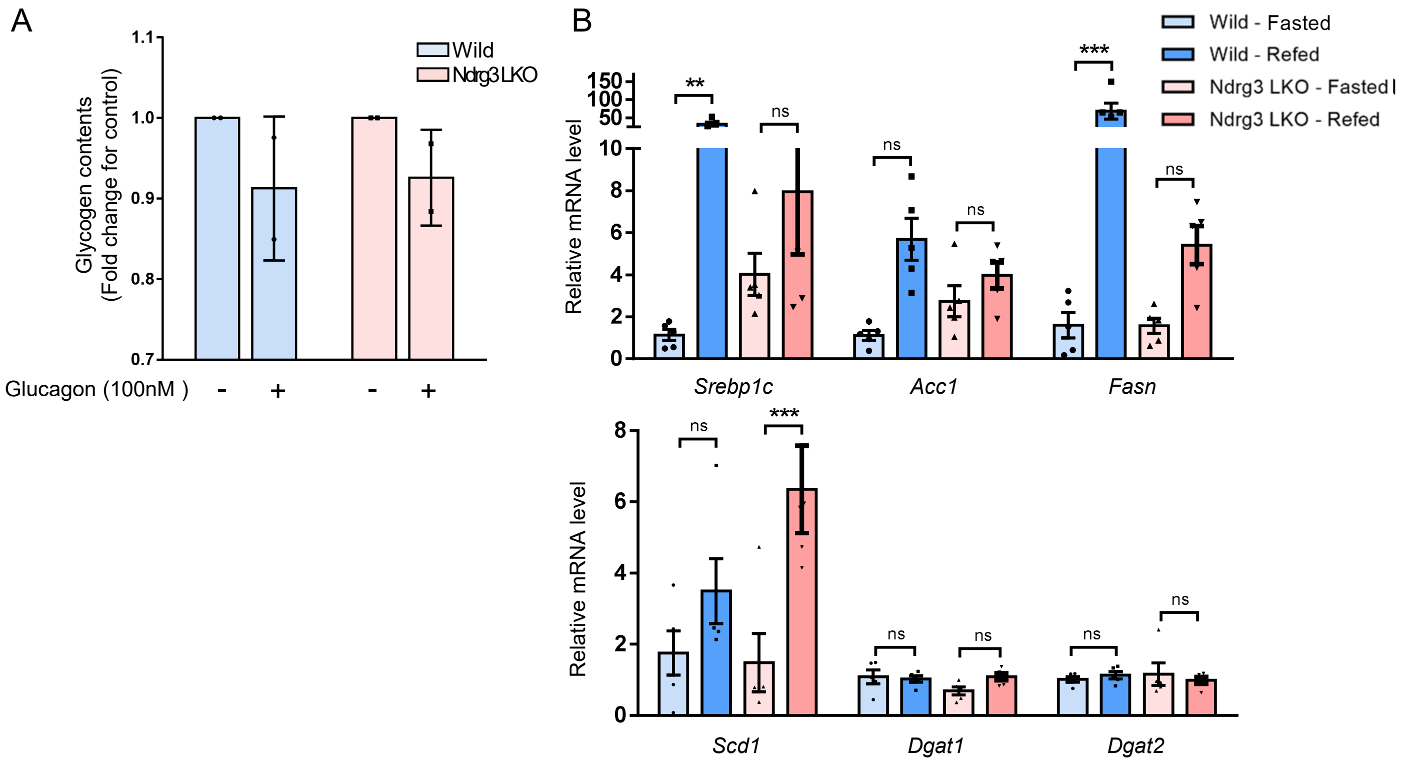

Figure S5. Glycogenolytic and lipogenic activities in Ndrg3 LKO mouse hepatocytes and livers. (A) Glycogen content measured in primary hepatocytes after stimulation with 100nM glucagon for 1 h. Two independent experiments were performed in duplicates. Data are presented as mean ± SD. (B) qRT-PCR analysis of lipogenic gene expression in wild-type and Ndrg3 LKO mouse livers at 2 months of age (n=5 per group). Gene expression was normalized using *Gapdh* as the reference. Data are presented as mean ± SEM. *p*-values were calculated using two-tailed Student's *t*-test (B). $^{**}p < 0.01$ and $^{***}p < 0.001$ vs wild mice.

Figure S6

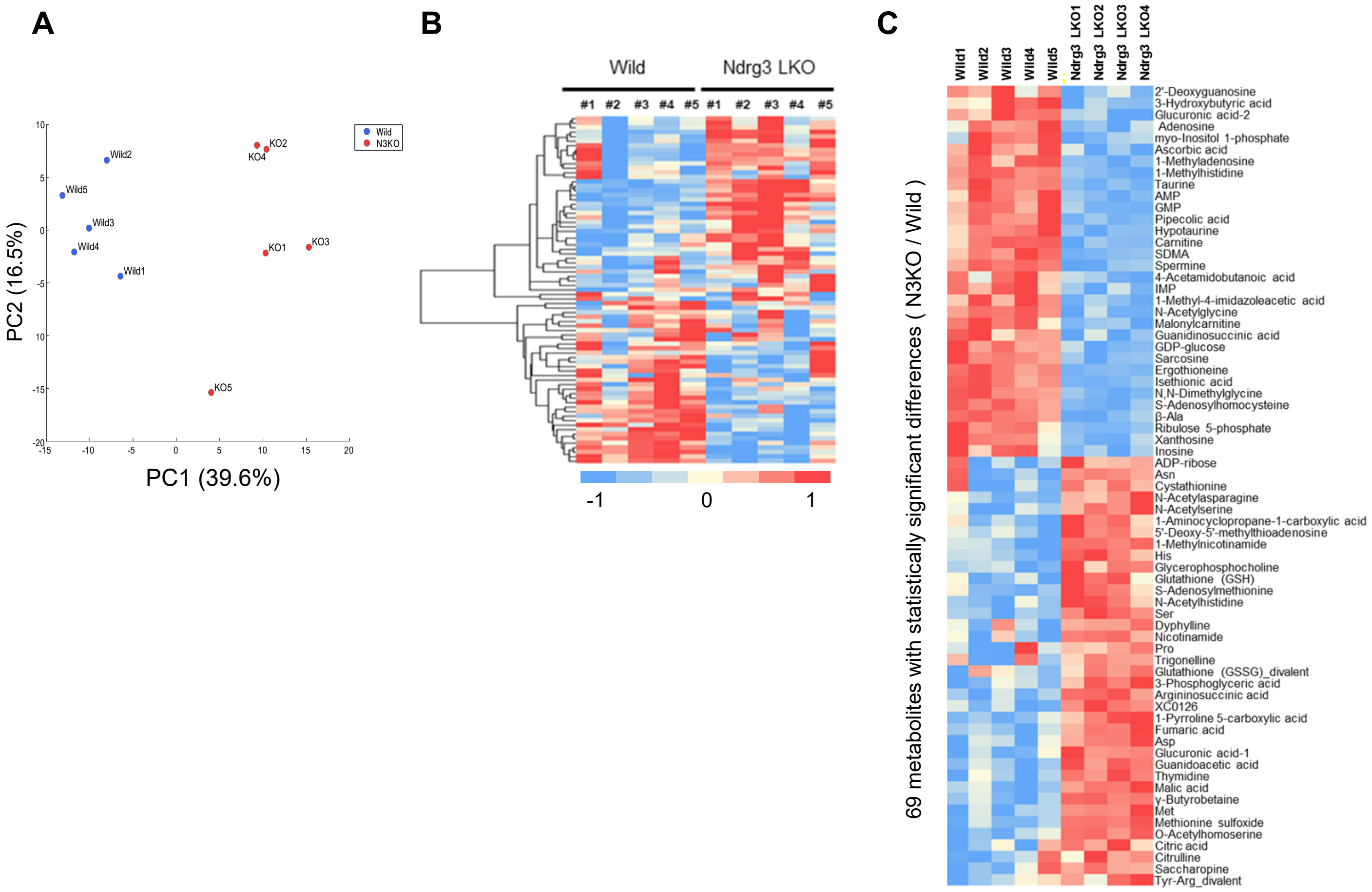

Figure S6. Metabolomic alterations in methionine cycle and branch pathways in Ndrg3 LKO livers. (A) Principal component analysis (PCA) based on the normalized metabolomic data for the livers of wild-type (n=5, blue dots) and Ndrg3 LKO mice (n=5, red dots). Percentage value of each axis in parentheses means the contribution rate for the first (PC1) and the second (PC2) principal components, and plot labels indicate sample name. Mice at 10-12 weeks of age were sacrificed under *ad libitum* states and used in the metabolome analysis. An outlier in Ndrg3 LKO group (KO #5) was found to have developed severe ascites, and was excluded from subsequent analyses. (B) One-way hierarchical clustering analysis (HCA) of metabolite peaks extracted from the analysis of liver tissues of wild-type (n=5) and Ndrg3 LKO (n=5) mice. Horizontal and vertical axes indicate sample labeling and metabolite peak, respectively. An outlier in Ndrg3 LKO group (KO #5) was found to have developed severe ascites, and was excluded from subsequent analyses. (C) Heatmap for 68 metabolites satisfying ($p<0.05$, $\log_2$(fold change) >1) between wild-type (n=5) and Ndrg3 LKO (n=4) mice. Red, relatively up-regulated. Blue, relatively down-regulated.

Figure S7

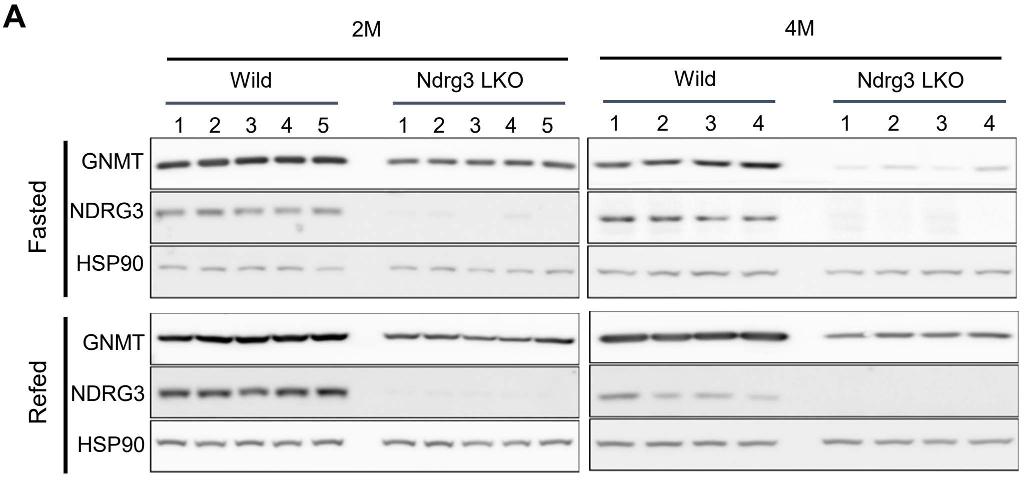

Figure S7. Dysregulation of GNMT expression in Ndrg3 LKO livers. (A) Western blot analysis of GNMT expression in wild-type and Ndrg3 LKO mouse livers at the age of 2 months (n=5 per group) or 4 months (n=4 per group). Mice were fasted for 15h or refed for additional 6h before being subject to the western blot analysis.

Table S1. List of primer sequences for quantitative real-time RT-PCR analysis and mouse genotyping.

Target gene	Species	Forward (5'→3')	Reverse (5'→3')
Primers for qRT-PCR			
Ndrg3 (exon4)	M. Musculus	GCATGGTATGGTCCACGTCA	CATCCATGGTGGGGTACTGG
Mat1a	M. musculus	CCTTCTCTGGAAAGGACTACACC	GACAGAGGTTCTGCCACACCAA
Mtap	M. musculus	CATTGCAGTGCCAGAGGAGTGT	TGAATGGCACCGAAGTCCTAGC
Pemt	M. musculus	ACTCATGCATGCTAGTCCCA	AGCAGTGAAGGGCTCTTCAT
Bhmt	M. musculus	GGAGGCTGCGCGGTTGAAA	CTAGTGGCAACTCGGGGTTCC
Gnmt	M. musculus	GCCAGACTGCAAAGGTGACCA	GTCGTAATGTCCTTGGTCAGGTCA
Ahcy	M. musculus	CAGCTTCTGTCAGGCATCCG	AACTTGCTCTTGGTGACAGAATCG
Cbs	M. musculus	CACAGTGCTGACCAAATCCCCC	CACACTTGGCCAAGAGCTCAC
Pygl	M. musculus	GGATTTTTGTGGACATTGAAAAA	AACAAGGCCACAATTCTGTCTAA
Agl	M. musculus	CCATTGCCTCTAAATTGACTTTG	ACCTCAAGTTTTCACAGAATGGA
Phka2	M. musculus	AGCCAAGATGAACTTGACCAGTA	GGGAGTCAACAAGATTCAGTGAC
Gbe1	M. musculus	TGAAGCCAATAAGACAATCACCT	CCAGTCTCTGATGACCTCCATAC
G6pt	M. musculus	TTGGAGTCATAGCCAATGAGAGT	AAGCAAGAAGAAGACAACTGTGC
Gaa	M. musculus	GCCCTACCTGTACACTCTCTTCC	AAGTAGCCCGTCACTTCAGTTTT
G6pc	M. musculus	ACCGACTACTACAGCAACAGCTC	ATGCGTTGGCTTTTTCTTTCCTC
Gapdh	M. musculus	AGGTCGGTGTGAACGGATTTG	TGTAGACCATGTAGTTGAGGTCA
Pck1	M. musculus	CTGGCACCTCAGTGAAGACA	TCGATGCCTTCCCAGTAAAC
G6pc	M. musculus	TCTGTCCCGGATCTACCTTG	GTAGAATCCAAGCGCGAAAC
Fbp1	M. musculus	CTGATATTCACCGCACTCTGG	CGGCCTTCTCCATGACATAAG
Srebp1c	M. musculus	GGAGCCATGGATTGCACATT	CAGGAAGGCTTCCAGAGAGG
Acc1	M. musculus	GCCTCTTCCTGACAAACGAG	TGACTGCCGAAACATCTCTG
Fasn	M. musculus	ACCTTAGAGGCAGTGCAGGA	GGAGCGCAGGATAGACTCAC
Scd1	M. musculus	CCGAAGAGGCAGGTGTAGAG	CCAAGAGATCTCCAGTTCT
Dgat1	M. musculus	GGCCCAAGGTAGAAGAGGAC	GATCAGCATCACCACACACC
Dgat2	M. musculus	AGGCCCTATTTGGCTACGTT	CTTCAGGGTGACTGCGTTCT
Rpl32	M. musculus	GGCCTCTGGTGAAGCCCAAGATCG	CCTCTGGGTTTCCGCCAGTTTCGC
Genotyping primers			
Ndrg3 KO	M. musculus	GAGATGGCGCAACGCAATTAAT	AGCTCAGTAGTACAGCACTTCCTTCG
Wild	M. musculus	GGAACTTCAGTTACAGATGGCTCT	CAGATTTATCTTTCACCCTTAAGCA
Alb-Cre	M. musculus	GAAGCAGAAGCTTAGGAAGATGG	TTGGCCCCTTACCATAACTG
Flp	M. musculus	GTGGATCGATCCTACCCCTTGCG	GGTCCAACTGCAGCCCAAGCTTCC
Genotyping primers for tissue specificity of *Ndrg3* KO			
Ndrg3 LKO	M. musculus	GATCTCTCAGTCTCAAACAC	AGCTCAGTAGTACAGCACTTCCTTCG

Table S2. Metabolite list for enriched metabolome sets satisfying ($p<0.05$, $\log_2$(fold change) >1) in the livers of Ndrg3 LKO mice(n=4) relative to wild type(n=5).

Up-regulated pathways and metabolites				
Term	($-\log_{10}$ (*p*-val))	Hits/Total	Raw *p*-val	Metabolites
Arginine and Proline Metabolism	4.58	9/53	2.60E-05	Argininosuccinic acid, Guanidoacetic acid, Fumaric acid, L-Proline, L-Aspartic acid, Citrulline, S-Adenosylhomocysteine, 1-Pyrroline-5-carboxylic acid, D-Proline
Aspartate Metabolism	3.16	6/35	0.0006	Argininosuccinic acid, L-Asparagine, L-Aspartic acid, Citrulline, D-Aspartic acid, Fumaric acid
Methionine Metabolism	2.66	6/43	0.0021	L-Cystathionine, L-Serine, L-Methionine, 5'-Methylthioadenosine, S-Adenosylmethionine, Methionine sulfoxide
Glycine and Serine Metabolism	2.63	7/59	0.0023	L-Cystathionine, Guanidoacetic acid, L-Serine, L-Methionine, 3-Phosphoglyceric acid, S-Adenosylmethionine, D-Serine
Methylhistidine Metabolism	2.21	2/4	0.0061	L-Histidine, S-Adenosylmethionine
Urea Cycle	1.87	4/29	0.0134	Argininosuccinic acid, Fumaric acid, L-Aspartic acid, Citrulline
Ammonia Recycling	1.72	4/32	0.0188	L-Asparagine, L-Histidine, L-Serine, L-Aspartic acid
Spermidine and Spermine Biosynthesis	1.71	3/18	0.0194	L-Methionine, 5'-Methylthioadenosine, S-Adenosylmethionine
Nicotinate and Nicotinamide Metabolism	1.51	4/37	0.0308	1-Methylnicotinamide, Adenosine diphosphate ribose, S-Adenosylmethionine, Niacinamide
Homocysteine Degradation	1.47	2/9	0.0333	L-Cystathionine, L-Serine
Down-regulated pathway and metabolites				
Term	($-\log_{10}$ (*p*-val))	Hits/Total	Raw *p*-val	Metabolites
Histidine Metabolism	2.47	7/43	0.0033	1-Methylhistidine, Adenosine monophosphate, Beta-Alanine, 3-Methylhistidine, S-Adenosylhomocysteine, Methylimidazole, Acetic acid
Methylhistidine Metabolism	2.13	2/4	0.0072	3-Methylhistidine, S-Adenosylhomocysteine
Methionine Metabolism	1.78	5/43	0.0165	Adenosine monophosphate, Adenosine, Dimethylglycine, Sarcosine, S-Adenosylhomocysteine
Betaine Metabolism	1.43	3/21	0.0369	Adenosine, Dimethylglycine, S-Adenosylhomocysteine
Carnitine Synthesis	1.37	3/22	0.0417	Ascorbic acid, L-Carnitine, S-Adenosylhomocysteine
Purine Metabolism	1.35	7/74	0.0446	Adenosine monophosphate, Adenosine, Deoxyguanosine, Inosinic acid, Inosine, Xanthosine, Guanosine monophosphate
